# Prediction of skin disease using a new cytological taxonomy based on cytology and pathology with deep residual learning method

**DOI:** 10.1038/s41598-021-92848-y

**Published:** 2021-07-02

**Authors:** Jin Bu, Yu Lin, Li-Qiong Qing, Gang Hu, Pei Jiang, Hai-Feng Hu, Er-Xia Shen

**Affiliations:** 1 Hospital for Skin Disease (Institute of Dermatology), Chinese Academy of Medical Sciences and Peking Union Medical College, Nanjing, 210042 Jiangsu China; 2 Guangzhou South China Biomedical Research Institute, Co., Ltd, Guangzhou, 510275 Guangdong China; 3 Nanxishan Hospital of Guangxi Zhuang Autonomous Region, Guilin, 541002 Guangxi China; 4 School of Agriculture, Sun Yat-Sen University, Guangzhou, 510275 Guangdong China; 5 Xinhua College of Sun Yat-Sen University, Guangzhou, 510520 Guangdong China; 6 School of Electronics and Information Technology (School of Microelectronics), Sun Yat-Sen University, Guangzhou, 510006 Guangdong China; 7 Sino-French Hoffmann Institute, School of Basic Sciences, The Second Affiliated Hospital of Guangzhou Medical University, State Key Laboratory of Respiratory Disease, Guangdong Provincial Key Laboratory of Allergy & Clinical Immunology, Guangzhou Medical University, Guangzhou, 511436 Guangdong China; 8 The State Key Laboratory of Respiratory Disease, The First Affiliated Hospital, Guangzhou Medical University, Guangzhou, 510182 Guangdong China

**Keywords:** Skin diseases, Computational science

## Abstract

With the development of artificial intelligence, technique improvement of the classification of skin disease is addressed. However, few study concerned on the current classification system of International Classification of Diseases, Tenth Revision (ICD)-10 on Diseases of the skin and subcutaneous tissue, which is now globally used for classification of skin disease. This study was aimed to develop a new taxonomy of skin disease based on cytology and pathology, and test its predictive effect on skin disease compared to ICD-10. A new taxonomy (Taxonomy 2) containing 6 levels (Project 2–4) was developed based on skin cytology and pathology, and represents individual diseases arranged in a tree structure with three root nodes representing: (1) Keratinogenic diseases, (2) Melanogenic diseases, and (3) Diseases related to non-keratinocytes and non-melanocytes. The predictive effects of the new taxonomy including accuracy, precision, recall, F1, and Kappa were compared with those of ICD-10 on Diseases of the skin and subcutaneous tissue (Taxonomy 1, Project 1) by Deep Residual Learning method. For each project, 2/3 of the images were included as training group, and the rest 1/3 of the images acted as test group according to the category (class) as the stratification variable. Both train and test groups in the Projects (2 and 3) from Taxonomy 2 had higher F1 and Kappa scores without statistical significance on the prediction of skin disease than the corresponding groups in the Project 1 from Taxonomy 1, however both train and test groups in Project 4 had a statistically significantly higher F1-score than the corresponding groups in Project 1 (*P* = 0.025 and 0.005, respectively). The results showed that the new taxonomy developed based on cytology and pathology has an overall better performance on predictive effect of skin disease than the ICD-10 on Diseases of the skin and subcutaneous tissue. The level 5 (Project 4) of Taxonomy 2 is better on extension to unknown data of diagnosis system assisted by AI compared to current used classification system from ICD-10, and may have the potential application value in clinic of dermatology.

## Introduction

A significant rise was demonstrated in the incidence of the majority of skin disease over the past decades^1^. Compared to disorders from other systems, diagnosis of skin disease is much more depended on lesion presentation, with more than 1500 different dermatological diagnoses, general practitioner diagnostic accuracy in dermatological disease has been estimated to be from 48 to 77%^2^, therefor the clinicians face a challenge to increase diagnostic accuracy and further improve theropy efficiency.

A lot of researches focused on the technique improvement of the diagnosis, especially on artificial intelligence^3^. Binder et al.^4^ used computerized image analysis and an artificial neural network to automatically diagnose pigmented skin lesions. The sensitivity and specificity of the computerized system were 90% and 74%, respectively.

Verma et al.^5^ classified erythemato-squamous diseases by ensemble 5 different data mining techniques, and the results showed that the proposed ensemble method generates more efficient use of the dataset and give more accurate rate than individual data mining techniques.

Sharma et al.^6^ compared Support Vector Machine and Artificial Neural Network, along with an ensemble of these two techniques for classification of erythemato-squamous diseases, and found that the ensemble model has achieved a remarkable performance with the highest accuracy.

Moradi and Mahdavi-Amiri^7^ propose a kernel sparse representation based method for segmentation and classification of melanoma images, and the evaluation results demonstrate their approach to be competitive as compared to the available state-of-the-art methods.

Yap et al.^8^ developed a multimodal classifier, which outperforms a baseline classifier that only uses a single macroscopic image in both binary melanoma detection and in multiclass classification.

Chang and Chen^9^ used decision tree of data mining combining with neural network classification methods to construct the best predictive model on six major skin diseases, and found that the neural network model had the highest accuracy in prediction.

The main work of these investigations is listed in Table 1. However, all of the investigations focused on improvement of diagnosis effects with the assistance of the artificial intelligence techniques, few researches concentrating on the imperfection of the current classification system of dermatology and venereology have been developed. The International Classification of Diseases, Tenth Revision (ICD)-10 is now globally universal in order to keep consistency in disease diagnosis, however, the literature on the shortcomings of the ICD-10 is scant. Recent studies have found deficiencies in the classification of allergic conditions by ICD-10 codes^10,11^, and a new revision ––ICD-11 ––is currently being developed with the aim of solving problems^12^.

**Table 1:** Investigations focusing on skin disease classification using artificial intelligence techniques.

Author	Year	Skin diseases	Imaging type	Method	Accuracy
Binder et al.^4^	1998	Pigmented skin lesions	Microscopy images	Computerized image analysis and an artificial neural network	The sensitivity and specificity of the computerized system were 90% and 74%, respectively
Verma et al.^5^	2019	Erythemato-squamous diseases	Dermatology database including macroscopic image; histopathological attribute; family history	An ensemble data mining based on 5 different data mining techniques, including Classification and Regression Trees, Support Vector Machines, Decision Tree, Random Forest and Gradient Boosting Decision Tree	The accurate rate was 98.64%
Sharma et al.^6^	2013	Erythemato-squamous diseases	Dermatology data	An ensemble data mining based on 2 different data mining techniques including Support Vector Machine and Artificial Neural Network	99.25% and 98.99% at training and testing stages respectively
Moradi and Mahdavi-Amiri^7^	2019	Erythemato-squamous diseases	Two benchmark dermoscopic datasets and one digital image dataset	A kernel sparse representation based method	﻿The method used by Moradi and Mahdavi-Amiri achieved the highest sensitivity as compared to the state-of-the-art methods on the PH2 dataset
Yap et al.^8^	2018	Melanoma	Dermatoscopic image, macroscopic image and patient metadata	Convolutional neural networks	The multimodal classifier outperforms a baseline classifier that only uses a single macroscopic image in both binary melanoma detection (AUC 0.866 vs 0.784) and in multiclass classification (mAP 0.729 vs 0.598)
Chang and Chen^9^	2009	Six major skin diseases	Skin disorder database including clinical and histopathological attributes	Decision tree of data mining combining with neural network classification methods	The neural network model and the sensitivity analysis combining with decision tree model have the highest accuracy (92.62%) and the least accuracy (80.33%) in prediction
Esteva et al.^15^	2017	Melanoma and skin cancers	Macroscopic images and dermoscopy images	Convolutional neural networks	The convolutional neural networks achieve performance on par with all tested experts across both tasks, demonstrating an artificial intelligence capable of classifying skin cancer with a level of competence comparable to dermatologists
This study	2020	Dermatology and venereology	Macroscopic images	Recurrent neural network	The new taxonomy is overall better on prediction of skin disease than the ICD-10 on Diseases of the skin and subcutaneous tissue

With in-depth researches on pathogenesis of skin disease, the knowledge on dermatology is improved and multiple diseases have been approved that their initial classifications are not accurate, for example, pyogenic granuloma sounds like an infectious diseases but actually is a kind of hemangioma, classification and nomenclature of vascular malformations have also changed^13^, and sebopsoriasis lacks a specific code^14^. So, the modern dermatology faces an imperious demand of classification with being more scientific. Esteva et al.^15^ developed a dermatologist-level system for skin cancer classification, although the aim of this study was to test an artificial intelligence capable of classifying skin cancer, it provides a direction to re-classify skin disease from different aspects.

Based on the above considerations, we conduct this study to develop a new taxonomy based on the cytology and pathology, and to further test the new taxonomy on diagnosis effects by Deep Residual Learning method, and compared with the ICD-10 on Diseases of the skin and subcutaneous tissue, in order to find a new classification benefiting prediction, having potential application in clinical practices in dermatology and venereology.

## Materials and methods

**Figure 1:**
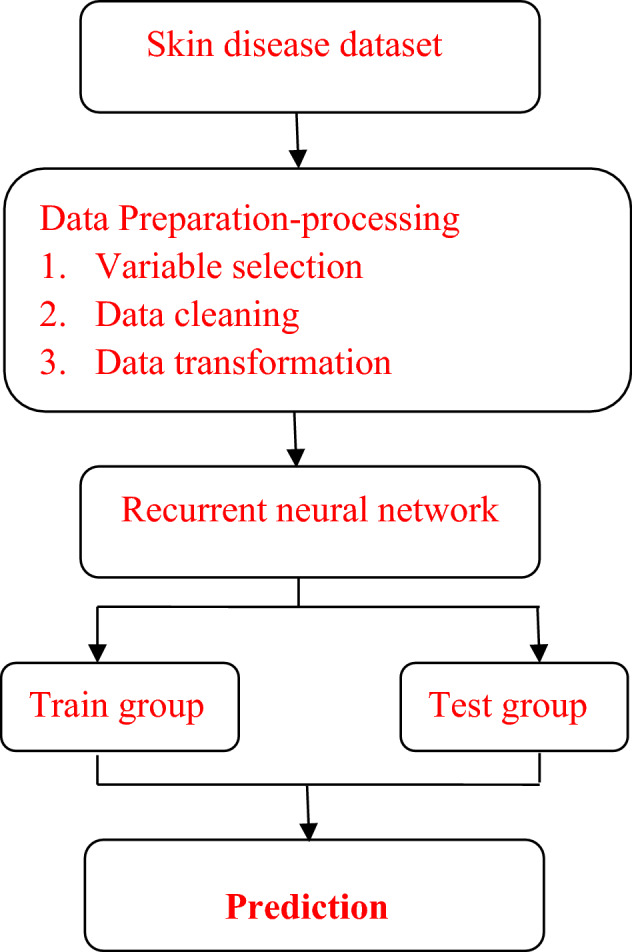
Methodological approach for skin diseases.

Figure 1 demonstrates the whole structure of methodology used in this research, and the approach used in this paper is completely data driven.

### Taxonomy

#### Taxonomy 1

ICD-10 Version: 2016—World Health Organization (http://apps.who.int/classifications/icd10/browse/2016/en).

#### Taxonomy 2

**Figure 2:**
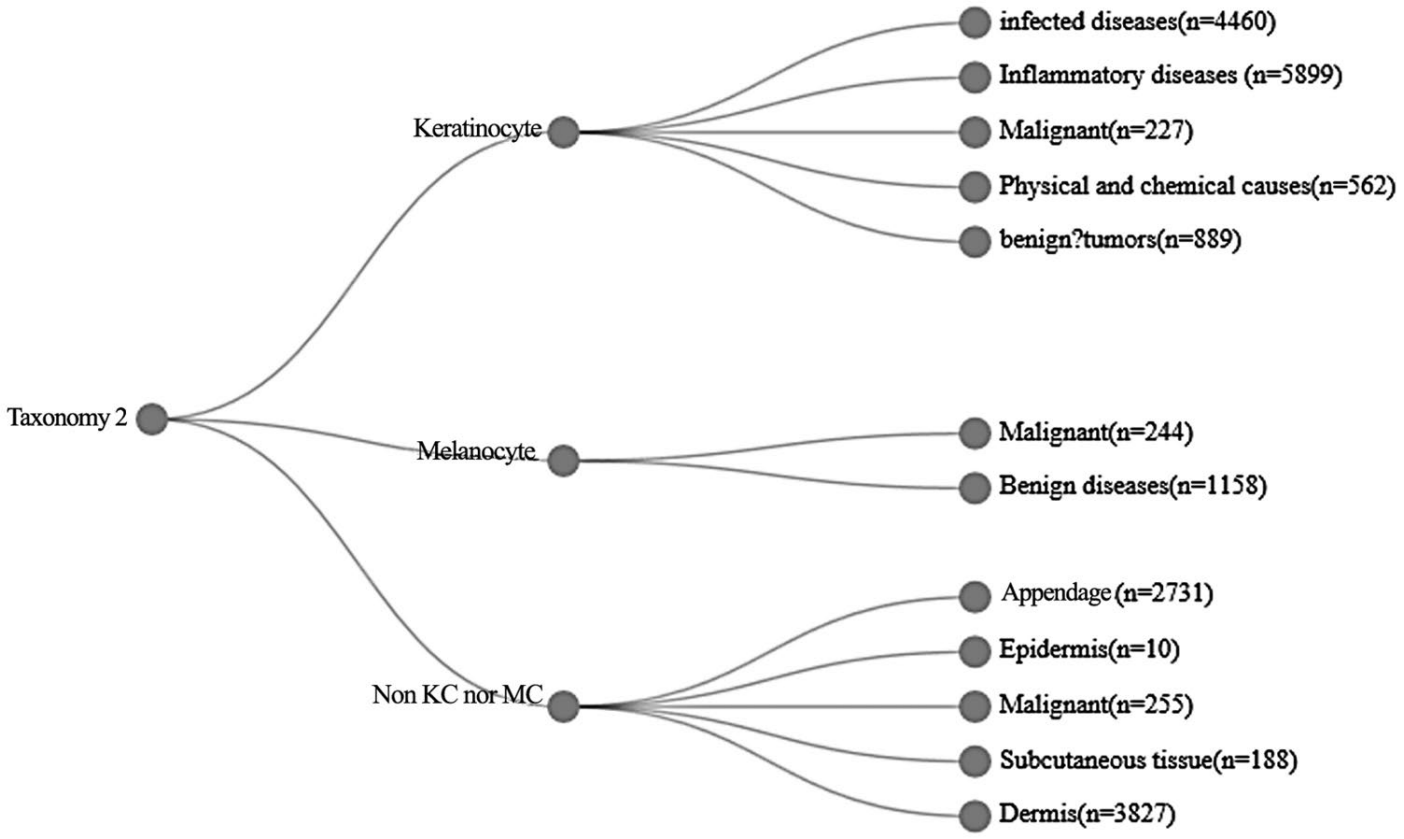
The first three levels contained by the taxonomy 2.

The taxonomy 2 represents 1,000 individual diseases arranged in a tree structure with three root nodes representing: (1) Keratinogenic diseases (KCs), (2) Melanogenic diseases (MCs), and (3) Diseases related to non-keratinocytes and non-melanocytes (Non-KC and non-MC). The taxonomy 2 was derived by dermatologists using a bottom-up procedure. Among the tree structure, individual diseases, initialized as leaf nodes, were merged based on organic or cellular similarity, until the entire structure was connected. The taxonomy 2 contains 6 levels, and the level 1–3 are present in Fig. 2. For each type of disease, a number indicates a different disease, and so on up to level 6.

The taxonomy is used in generating training classes that are both well-suited for machine learning classifiers and medically relevant. The root nodes are used in the first validation strategy and represent the source cell/organization of disease. The children of the root nodes (for example, malignant melanocytic lesions) are used in the second validation strategy, and represent disease classes that have similar clinical treatment plans.

### Projects setting

All images come from the following public databases, Atlas (http://www.atlasdermatologico.com.br/), Dermatoweb (http://www.dermatoweb.net/), dermnet (http://www.dermnet.com/), Dermnetnz (https://www.dermnetnz.org/), Emedicinehealth (https://www.emedicinehealth.com/), Globalskinatlas (http://www.globalskinatlas.com/), Meddean (http://www.meddean.luc.edu/), Uiowa (https://medicine.uiowa.edu/). A total of 56,571 images were collected. The acquisition program generates a list of images with classification tags for each website, downloads the corresponding images, and obtains a picture library with a description of the classification tags.

Taxonomy 1 was defined as Project 1. Finally, based on the resources of the image library, which should be balanced in two taxonomies, 11 classes were selected as project 1, including pemphigus, lichen planus, congenital ichthyosis, other dermatitis, pediculosis, scabies, herpes viral infections, unspecified viral infection, gonococcal infection, other sexually transmitted diseases, other congenital malformations of skin, and not elsewhere classified.

Level 3 from Taxonomy 2 is defined as Project 2, and contains a total of 2 classes: Inflammatory diseases; Infectious diseases. Level 4 from Taxonomy 2 is defined as Project 3, and contains a total of 4 classes: Virus, Parasite, Bacteria, Dermatitis. Level 5 from Taxonomy 2 is defined as Project 4, and contains a total of 11 categories: porokeratosis; herpes, simple genital; lichen planus; condilomas acuminados; ichthyosis; viral exanthems; pediculosis pubis; pemphigus; gonorrhea; eczema; sarna noruega.

### Data processing instructions

According to the Taxonomy 2, finally 1,847 images were extracted. And then, the images are screened to ensure that the two taxonomies contain the same ones, and finally a total of 1,160 images were obtained.

### Predictive model evaluation by recurrent neural network

After annotation of the images, our predictions on the two taxonomies are based on Deep Residual Learning for Image Recognition (deep learning), which belongs to CNN. For fair comparison, we adopt ResNet-50 pre-trained on ImageNet as the feature extraction network. Specifically, SGD optimizer with momentum 0.9 and weight decay 5e-4 is adopted, the initial learning rate is set as 1e-4. The batch size is set to 64 and the drop-out rate is 0.5.

Identify the images according to the Taxonomy 1: Project_1 represents the specific information of each picture marked using taxonomy1 classification system. Entity_id is the unique ID of the picture. Code_1 represents the number of images in each category under images marked with the taxonomy1 classification system. code_id is the category unique ID.

Identify the images according to the Taxonomy 2 (3–5 levels): Project 2, Project 3, Project 4 represents the specific information of each picture marked at the 3, 4, 5 level using the Taxonomy 2 system, respectively. entity_id is the unique ID of the picture. And code_2 represents the Taxonomy 2 system. At the 2, 3, 4, level under the marked images, respectively, the number of images in each category. code_id is the category unique ID.

For each project, 2/3 of the images were included as the training group, and the rest 1/3 of the images acted as the test group according to the category (class) as the stratification variable.

The accuracy, Kappa coefficient, Precision, Recall, and *F*1-score were calculated and compared between the two taxonomies.

Formulas:

$$ \begin{aligned}    & P{\text{recision}} = TP/\left( {TP + FP} \right) \\     & R{\text{ecall}} = TP/\left( {TP + FN} \right) \\     & {\text{F1-score}} = 2 \times P \times R/\left( {P + R} \right) \\  \end{aligned} $$

TP indicates the number of correct predictions for this category in the real classification, FP indicates the number of false predictions in this category for unreal classification, FN indicates that the number of this category is not correctly predicted in the real classification.

## Results

### The overall comparison on predicted results between projects

Table 2 showed the comparison of the predicted results of projects by different categories. Only the Project 4 has a higher accuracy on prediction of skin disease.

**Table 2:** Comparison of the identified results of projects by different categories.

Project	Groups	Accuracy (%)	ave_PPV_ precision (%)	ave_TPR_ recall (%)	ave_F1 (%)	Kappa (%)
Project_1	Train	99.52	98.01 ± 2.97	90.77 ± 13.40	93.63 ± 7.99	95.62
	Test	97.73	88.51 ± 10.74	70.99 ± 26.44	74.43 ± 21.89	79.08
Project_2	Train	98.56	98.12 ± 1.05	97.41 ± 2.73	97.76 ± 1.90	95.52
	Test	95.69	94.28 ± 3.22	92.17 ± 8.37	93.17 ± 5.85	86.35
Project_3	Train	99.15	98.09 ± 2.91	96.22 ± 4.01	97.12 ± 3.06	95.04
	Test	96.59	86.42 ± 7.20	83.56 ± 9.69	84.81 ± 7.63	80.42
Project_4	Train	99.90	99.46 ± 1.58	97.97 ± 4.39	98.67 ± 2.63	99.13
	Test	99.45	96.22 ± 5.27	89.81 ± 15.95	91.88 ± 9.59	95.10
Project_1 VS Project_2 P value	Train	0.031	0.755	0.921	0.374	
	Test	0.008	0.481	0.093	0.138	
Project_1 VS Project_3 P value	Train	0.400	1.000	0.792	0.471	
	Test	0.127	0.727	0.647	0.471	
Project_1 VS Project_4 P value	Train	0.188	0.177	0.058	0.025*	
	Test	0.008	0.089	0.016*	0.005**	

*PPV* positive predictive value, *TPR* true positive rate.

Except for the test group in Project 3, all of the train and test groups in the Projects (2,3, and 4) from Taxonomy 2 have a higher precision on prediction of skin disease than the corresponding group in the Project 1 from Taxonomy 1, while no differences are significant. For the recall rate of Projects, both train and test groups in the Projects (2,3, and 4) from Taxonomy 2 are better than the corresponding group Project 1 from Taxonomy 1, while only the test group in Project 4 has a statistically significantly higher recall rate than the test group in Project 1 (*P* = 0.016).

For the F1-score, both train and test groups in the Projects (2, 3, and 4) from Taxonomy 2 are better than the corresponding groups in Project 1 from Taxonomy 1, and both the train and test groups in Project 4 have a statistically significantly higher F1-score than the corresponding groups in Project 1 (*P* = 0.025 and 0.005, respectively).

All of the train and test groups in the Projects (2, 3, and 4) from Taxonomy 2 have a higher Kappa value on prediction of skin disease than the corresponding groups in the Project 1 from Taxonomy 1.

### Comparisons among classes in Projects

The results showed that all of the parameters including sensitivity and recall, specificity, positive predictive value (PPV) and precision, negative predictive value (NPV), and F1 in the 11 diseases of the train groups are all better than those in the test group in Project 1 (Table 3). And the F1 in part of diseases, especially of gonococcal infection and Herpes viral infections, in the test group are much lower compared with that in the train group.

**Table 3:** Effects of AI prediction between train and test groups among various diseases in Project 1 (%).

	Classes	1	2	3	4	5	6	7	8	9	10	11
Train group	Sensitivity and recall	96.00	57.14	89.47	96.97	89.74	94.59	75.00	100.00	100.00	99.57	100.00
	Specificity	100.00	100.00	100.00	99.57	99.86	99.86	100.00	100.00	99.44	96.30	100.00
	PPV and precision	100.00	100.00	100.00	95.52	97.22	97.22	100.00	100.00	90.48	97.66	100.00
	NPV	99.72	99.60	99.73	99.71	99.45	99.72	99.60	100.00	100.00	99.31	100.00
	F1	97.96	72.73	94.44	96.24	93.33	95.89	85.71	100.00	95.00	98.60	100.00
Test group	Sensitivity and recall	76.92	25.00	90.00	76.47	80.95	70.00	14.29	85.71	85.71	95.83	80.00
	Specificity	97.59	100.00	99.74	99.18	99.47	99.74	100.00	100.00	98.15	84.38	99.49
	PPV and precision	68.97	100.00	90.00	89.66	89.47	93.33	100.00	100.00	72.00	90.20	80.00
	NPV	98.38	99.25	99.74	97.84	98.95	98.44	98.50	99.75	99.20	93.10	99.49
	F1	72.73	40.00	90.00	82.54	85.00	80.00	25.00	92.31	78.26	92.93	80.00

1: Other congenital malformations of skin; 2: Gonococcal infection; 3. Other dermatitis; 4. Unspecified viral infection characterized by skin and mucous membrane lesions; 5. Pemphigus; 6. Congenital ichthyosis; 7. Herpes viral [herpes simplex] infections; 8. Scabies; 9. Other predominantly sexually transmitted diseases, not elsewhere classified; 10. Lichen planus; 11. Pediculosis and phthiriasis; *PPV* positive predictive value, *NPV* negative predictive value.

While the results showed that all of the parameters including sensitivity and recall, specificity, PPV and precision, NPV, and F1 in the 11 diseases of the train groups are similar with those in the test group at different classification levels in Projects 2–4 of Taxonomy 2 (Project 2/Level 3, Table 4; Project 3/Level 4, Table 5; Project 4/Level 5, Table 6).

**Table 4:** Effects of AI prediction between train and test groups among various diseases in Project 2 (%).

	Classes	Infectious diseases	Inflammatory diseases
Train group	Sensitivity and recall	95.48	99.34
	Specificity	99.34	95.48
	PPV/precision	97.37	98.86
	NPV	98.86	97.37
	F1	96.42	99.10
Test group	Sensitivity/recall	86.25	98.09
	Specificity	98.09	86.25
	PPV/precision	92.00	96.55
	NPV	96.55	92.00
	F1	89.03	97.31

*PPV* positive predictive value, *NPV* negative predictive value.

**Table 5:** Effects of AI prediction between train and test groups among various diseases in Project 3(%).

Group	Classes	Virus	Parasite	Bacteria	Dermatitis
Train group	Sensitivity/recall	92.42	100.00	93.10	99.34
	Specificity	99.43	100.00	100.00	94.16
	PPV/precision	93.85	100.00	100.00	98.53
	NPV	99.28	100.00	99.44	97.32
	F1	93.13	100.00	96.43	98.94
Test group	Sensitivity/recall	85.29	75.00	77.42	96.51
	Specificity	97.79	99.47	98.90	83.95
	PPV/precision	78.38	85.71	85.71	95.90
	NPV	98.61	98.95	98.10	86.08
	F1	81.69	80.00	81.36	96.20

*PPV* positive predictive value, *NPV* negative predictive value.

**Table 6:** Effects of AI prediction between train and test groups among various diseases in Project 4 (%).

	Classes	1	2	3	4	5	56	7	8	9	10	11
Train group	Sensitivity/ recall	100.00	100.00	99.78	100.00	100.00	100.00	100.00	97.44	85.71	94.74	100.00
	Specificity	100.00	100.00	98.99	100.00	100.00	100.00	100.00	100.00	100.00	99.86	100.00
	PPV/precision	100.00	100.00	99.35	100.00	100.00	100.00	100.00	100.00	100.00	94.74	100.00
	NPV	100.00	100.00	99.66	100.00	100.00	100.00	100.00	99.86	99.87	99.86	100.00
	F1	100.00	100.00	99.57	100.00	100.00	100.00	100.00	98.70	92.31	94.74	100.00
Test group	Sensitivity /recall	84.62	100.00	99.58	100.00	100.00	97.06	90.00	95.24	50.00	100.00	71.43
	Specificity	100.00	99.75	96.25	99.74	100.00	100.00	100.00	99.21	100.00	99.74	100.00
	PPV/ precision	100.00	87.50	97.55	95.45	100.00	100.00	100.00	86.96	100.00	90.91	100.00
	NPV	98.94	100.00	99.35	100.00	100.00	99.73	99.74	99.73	99.50	100.00	99.49
	F1	91.67	93.33	98.56	97.67	100.00	98.51	94.74	90.91	66.67	95.24	83.33

1: Pediculosis and phthiriasis; 2: herpes simple genital; 3. lichen planus; 4: condilomas acuminados; 5. Ichthyosis; 6. viral exanthems; 7: pediculosis pubis; 8. pemphigus ; 9: Gonorrhea; 10: eczema; 11: sarna noruega; *PPV* positive predictive value, *NPV* negative predictive value.

## Discussion

Descriptive dermatology of the morphological phenomena of skin has been developed for more than two thousand years^16^. Briefly, our ancestors have separated skin disorders, depending either on their location, their appearance or more interestingly their suspected cause. In consequence, the textbooks, that have fashioned our education, have also adopted sometimes very different ways to present and classify skin diseases^17^. Classification by similarities became more and more difficult as the complexity of disease was realized^18^. New classification which may help diagnosis, disease management, and discipline development is in urgent need.

This study developed a new taxonomy (Taxonomy 2) containing 6 levels (project 2–4) of most skin disease based on cytology and pathology, which is a completely new work on the dermatology and venereology compared to the previous work focusing on classification of one type or several skin disease by AI techniques^4–9^.

In order to investigate the predictive effect of the new taxonomy on skin disease, we further compared the accuracy, precision, recall, F1, and Kappa of the new taxonomy with the ICD 10 using Deep Residual Learning method. Precision, recall, and F1-score are commonly used to evaluate the predictive effect of models/projects in multi-class prediction. Precision is the number of correctly predicted samples divided by the number of all samples, that is, the prediction accuracy rate of the model, and is used to measure the proportion of correct discrimination among all predicted categories, similar to sensitivity. Recall is used to measure the proportion of correctly identified in all true categories, similar to specificity. The two constitute a pair of contradictory measures. F1 score is used to weigh these two indicators. Deep CNNs has a potential widely application for diagnosis of skin diseases, with a higher accuracy compared with human dermatologists^19,20^, that is why we applied it to prediction diseases based on different taxonomies, at same time to avoid instability of human beings.

Our results confirmed that the new taxonomy had a better performance in all parameters, and the final level of classification had a significant higher F1-score than the ICD-10 taxonomy, which means it may be better on extension to unknown data and may provide a better taxonomy system for skin disease prediction under assistance of AI techniques in the future.

The literature on the shortcomings of the ICD-10 is very few. A compatible version of the ICD-10 specifically adapted to dermatology was produced in Spain in 1999 to overcome these shortcomings. González-López et al.^21^ confirmed that the ICD-10 system does have some minor shortcomings when it comes to coding certain diseases, particularly newly discovered and emerging diseases. A classification of hypersensitivity/allergic diseases was constructed to validate it for ICD-11 by crowdsourcing the allergist community^11^, because the well-known misclassification and/or under-notification of these diseases in the ICD, which has a direct and huge detrimental impact on hypersensitivity/allergic diseases data^22^. However, a reclassification of whole disciplinary systems of dermatology hasn’t been tried yet, so we attempted to construct a new taxonomy in this study. The results of current study confirmed that the taxonomy 2 developed has advance on the disease prediction compared to ICD-10 on skin diseases, which may have a potential application value in future clinical practice in dermatology and venereology.

The current study has the following limitations: 1. AI is the only detection technology for comparison, but is not the gold standard for prediction, so it has system error, which may affect the comparison result. 2. The dermatological data didn’t include histopathological images, and it may influence accurate classification effect. 3. The train and test groups of Project 1 have differences on all of the three parameters. And the Project 3 and Project 4 have a difference on precision and F1-score, respectively. Our purpose of dividing the images into 2 groups is to prevent model overfitting, which means that it performs well in the training group, but may be very poor when it is changed to other data and cannot be well predicted. We used 2/3 of the data to build the model and adjust the parameters in order to build a good model, however the difference between train and test groups indicate a low credibility of the results, the images of different types of diseases are not balanced, which may result from the not good enough quality of images of skin diseases, especially for some types.

## Conclusion and future work

In conclusion, this study is a try for dermatology precise or effective classification for discipline development, and this new taxonomy based on cytology and pathology we developed is an innovation and challenge for current dermatology classification from ICD-10, and has been provided to have an overall better performance on predictive effect including sensitivity and recall, specificity, PPV and precision, NPV, and F1, compared with ICD-10. The new taxonomy has the potential application value for clinical practice using AI techniques for skin prediction. However, a coming comprehensive system covering more skin disease and having different data including dermoscopic and histopathogical images are necessary for further confirmation of the stability of the taxonomy.

## Data Availability

The data that support the findings of this study are available from the first author (Jin Bu, dr.jinbu@gmail.com) upon reasonable request.
