# Author Correction: Prediction of skin disease using a new cytological taxonomy based on cytology and pathology with deep residual learning method

**DOI:** 10.1038/s41598-023-34103-0

**Published:** 2023-04-26

**Authors:** Jin Bu, Yu Lin, Li-Qiong Qing, Gang Hu, Pei Jiang, Hai-Feng Hu, Er-Xia Shen

**Affiliations:** 1 Hospital for Skin Disease (Institute of Dermatology), Chinese Academy of Medical Sciences and Peking Union Medical College, Nanjing, 210042 Jiangsu China; 2 Guangzhou South China Biomedical Research Institute, Co., Ltd, Guangzhou, 510275 Guangdong China; 3 Nanxishan Hospital of Guangxi Zhuang Autonomous Region, Guilin, 541002 Guangxi China; 4 School of Agriculture, Sun Yat-Sen University, Guangzhou, 510275 Guangdong China; 5 Xinhua College of Sun Yat-Sen University, Guangzhou, 510520 Guangdong China; 6 School of Electronics and Information Technology (School of Microelectronics), Sun Yat-Sen University, Guangzhou, 510006 Guangdong China; 7 Sino-French Hoffmann Institute, School of Basic Sciences, The Second Affiliated Hospital of Guangzhou Medical University, State Key Laboratory of Respiratory Disease, Guangdong Provincial Key Laboratory of Allergy & Clinical Immunology, Guangzhou Medical University, Guangzhou, 511436 Guangdong China; 8 The State Key Laboratory of Respiratory Disease, The First Affiliated Hospital, Guangzhou Medical University, Guangzhou, 510182 Guangdong China

Correction to: *Scientific Reports* https://doi.org/10.1038/s41598-021-92848-y, published online 02 July 2021

The original version of this Article contained an error in the Funding section.

“This work was supported by the National Natural Science Foundation of China (No. 81971482), the Science and Technology Planned Project of Bureau of Education of Guangzhou (No. 1201610221), the Science and Technology Program of Guangzhou, China (No.007095050049).”

now reads:

“This work was supported by the National Natural Science Foundation of China (No. 81971482), the Science and Technology Planned Project of Bureau of Education of Guangzhou (No. 1201610221), the Science and Technology Program of Guangzhou, China (No.202102080341).”

The original Article has been corrected.

